# Differential expression of major histocompatibility complex class I in developmental glioneuronal lesions

**DOI:** 10.1186/1742-2094-10-12

**Published:** 2013-01-24

**Authors:** Avanita S Prabowo, Anand M Iyer, Jasper J Anink, Wim GM Spliet, Peter C van Rijen, Eleonora Aronica

**Affiliations:** 1 Department of (Neuro)Pathology, Academic Medical Center, University of Amsterdam, Meibergdreef 9, Amsterdam, 1105 AZ, The Netherlands; 2 Departments of Pathology and Neurosurgery /Rudolf Magnus Institute for Neuroscience, University Medical Center Utrecht, Utrecht, The Netherlands; 3 SEIN – Stichting Epilepsie Instellingen Nederland, Heemstede, The Netherlands; 4 Swammerdam Institute for Life Sciences, Center for Neuroscience, University of Amsterdam, Amsterdam, The Netherlands

**Keywords:** Focal cortical dysplasia, Ganglioglioma, Cortical tubers, Neurons, Microglia, Major histocompatibility complex (MHC) class I

## Abstract

**Purpose:**

The expression of the major histocompatibility complex class I (MHC-I) in the brain has received considerable interest not only because of its fundamental role in the immune system, but also for its non-immune functions in the context of activity-dependent brain development and plasticity.

**Methods:**

In the present study we evaluated the expression and cellular pattern of MHC-I in focal glioneuronal lesions associated with intractable epilepsy. MHC-I expression was studied in epilepsy surgery cases with focal cortical dysplasia (FCD I, *n* = 6; FCD IIa, *n* = 6 and FCD IIb, *n* = 15), tuberous sclerosis complex (TSC, cortical tubers; *n* = 6) or ganglioglioma (GG; *n* = 15) using immunocytochemistry. Evaluation of T lymphocytes with granzyme-B^+^ granules and albumin immunoreactivity was also performed.

**Results:**

All lesions were characterized by MHC-I expression in blood vessels. Expression in both endothelial and microglial cells as well as in neurons (dysmorphic/dysplastic neurons) was observed in FCD II, TSC and GG cases. We observed perivascular and parenchymal T lymphocytes (CD8^+^, T-cytotoxic) with granzyme-B^+^ granules in FCD IIb and TSC specimens. Albumin extravasation, with uptake in astrocytes, was observed in FCD IIb and GG cases.

**Conclusions:**

Our findings indicate a prominent upregulation of MHC-I as part of the immune response occurring in epileptogenic glioneuronal lesions. In particular, the induction of MHC-I in neuronal cells appears to be a feature of type II FCD, TSC and GG and may represent an important accompanying event of the immune response, associated with blood–brain barrier dysfunction, in these developmental lesions.

## Introduction

Recent clinical and neuropathological evidence supports the critical role of a sustained inflammatory reaction in glioneuronal lesions with activation of both the innate and the adaptive immune response and involvement of different inflammatory pathways (for reviews see [1-4]). Interestingly, proinflammatory molecules have been shown to alter neuronal excitability and, in experimental models, to decrease the seizure threshold, contributing to neuronal cell death [2-4].

According to the current histopathological classification system [5], focal cortical dysplasia (FCD) has been classified into type I, characterized by cortical dyslamination (with three FCD subtypes according to the pattern of dyslamination), and type II, characterized by additional cytological abnormalities (FCD IIa with dysmorphic neurons and FCD IIb with dysmorphic neurons and balloon cells).

In a recent study [6], we confirmed the occurrence of complex inflammatory changes (involving both glial and neuronal cells) in FCD specimens and demonstrated that the severity of these changes is greater in FCD IIb than in specimens from patients with FCD I. The activation of components of the adaptive immunity, with the presence of T lymphocytes (CD8^+^, T-cytotoxic/suppressor immunophenotype), has also been mainly observed in FCD IIb specimens [6].

Whether these inflammatory changes represent a feature common to different developmental glioneuronal lesions and whether induction of major histocompatibility complex (MHC) class I molecules may be involved still needs to be clarified.

MHC-I molecules play a fundamental role in the immune system, in particular in the context of the adaptive immune response, but have also been shown to have non-immune functions, being involved in the regulation of activity-dependent brain development and plasticity (for a review see [7]). Interestingly, glial and neuronal MHC-I upregulation has been observed in brain specimens from patients with Rasmussen’s encephalitis (a severe inflammatory epileptic encephalopathy of childhood) and intractable epilepsy and it has been suggested that it plays a critical role in antigen-specific cytotoxicity [8,9].

The aim of the present study was to determine whether MHC-I is induced in focal glioneuronal lesions associated with intractable epilepsy. We evaluated the cellular distribution of MHC-I within a large spectrum of glioneuronal lesions, including focal cortical dysplasia type I, type IIa and type IIb, cortical tubers from patients with tuberous sclerosis complex (TSC) and gangliogliomas (GGs). Additionally, the presence of T lymphocytes with granzyme-B^+^ granules and albumin immunoreactivity within the lesion and its uptake into astrocytes were also analyzed.

## Material and methods

### Patients and controls

We examined a total of 48 surgical specimens: 6 FCD I (3, Ia; 3, Ib), 6 FCD IIa, 15 FCD IIb, 6 cortical tubers from patients with TSC and 15 GG. The cases included in this study were obtained from the departments of neuropathology of the Academic Medical Center (University of Amsterdam, UvA) in Amsterdam and the University Medical Center in Utrecht (UMCU), the Netherlands. The clinical characteristics derived from the patients’ medical records are summarized in Table 1. Patients underwent therapeutic surgical resection for refractory epilepsy and had, predominantly, medically intractable complex partial seizures. The postoperative seizure outcome was classified according to Engel [10]. All the patients included in our series did not have any apparent seizure activity in the 24 h before surgery. Patients who underwent implantation of strip and/or grid electrodes for chronic subdural invasive monitoring before resection were excluded from this study.

**Table 1:** Summary of clinical findings for epilepsy patients and controls

**Pathology type**	**Number of cases**	**Gender (males/females)**	**Mean age at surgery (years/range)**	**Localization**	**Mean duration of epilepsy (years/range)**
FCD I	6	3/3	27.8 (19 to 39)	4 Temporal	7.7 (6 to 13)
				2 Frontal	
FCD IIa	6	3/3	24.3 (11 to 24)	3 Frontal	6.1 (2 to 11)
				3 Temporal	
FCD IIb	15	7/8	23.2 (17 to 41)	8 Temporal	7.8 (3 to 15)
				7 Frontal	
Cortical tubers (TSC)	6	3/3	17.8 (5 to 35)	3 Frontal	13.5 (2.8 to 34)
				3 Temporal	
				2 Parietal	
GG	15	8/7	29.2 (15 to 46)	Temporal	7.8 (3 to 15)
Controls (no epilepsy)	6	3/3	30.1 (20 to 41)	Temporal	-

FCD, focal cortical dysplasia; TSC, tuberous sclerosis complex; GG, ganglioglioma.

To grade the degree of FCD, we followed the international consensus classification recently proposed [5]. All patients with cortical tubers fulfilled the diagnostic criteria for TSC [11]. For GG, we used the revised WHO classification of tumors of the central nervous system [12]. In five patients (one FCD, one TSC and three GG) a significant amount of perilesional tissue (normal-appearing cortex/white matter adjacent to the lesion) was resected as well. Brain tissue from patients with viral encephalitis (rabies encephalitis [13]; herpes simplex encephalitis (female, age at autopsy: 69 years) and Rasmussen’s encephalitis (*n* = 6; 4 females and 2 males; mean age at surgery: 20.6 years, range: 16 to 26) were also examined as positive controls. In addition, normal-appearing control cortex/white matter was obtained at autopsy from six young adult control patients (Table 1), without a history of seizures or other neurological diseases. All autopsies were performed within 12 h after death. Informed consent was obtained for the use of brain tissue. Tissue was obtained and used in a manner compliant with the Declaration of Helsinki.

### Tissue preparation

Formalin-fixed, paraffin-embedded tissue samples (one representative paraffin block per case containing the complete lesion or the largest part of the lesion resected at surgery) were sectioned at 6 μm and mounted on pre-coated glass slides (Star Frost, Waldemar Knittel GmbH, Braunschweig, Germany). Sections of all specimens were processed for hematoxylin eosin, luxol fast blue and Nissl stains as well as for immunocytochemical stainings for a number of neuronal and glial markers, which are described below.

### Immunocytochemistry

The primary antibodies used in the study are summarized in Table 2. Single-label immunocytochemistry was developed using the Powervision kit (Immunologic, Duiven, the Netherlands). 3,3-diaminobenzidine (Sigma, St Louis, USA) was used as the chromogen. Sections were counterstained with hematoxylin.

**Table 2:** Immunocytochemistry: primary antibodies

**Antigen**	**Primary antibody**	**Source**	**Dilution**
Glial fibrillary acidic protein (GFAP)	Polyclonal rabbit	DAKO, Glostrup, Denmark	1:4,000
Neuronal nuclear protein (NeuN)	Mouse clone MAB377	Chemicon, Temecula, CA, USA	1:2,000
HLA-DP, DQ, DR (MHC-II)	Mouse clone CR3/43	DAKO, Glostrup, Denmark	1:1000
HLA-A, B, C (MHC-I)	Mouse clone HC-10	Gift from Prof. J. Neefjes, NKI, the Netherlands	1:200
Vimentin	Mouse clone V9	DAKO, Glostrup, Denmark	1:400
Neurofilament (NF)	Mouse clone SMI311	Sternberger Monoclonals, Lutherville, MD, USA	1:1,000
Microtubule associated protein (MAP2)	Mouse clone HM2	Sigma,St. Louis, IL, USA	1:100
Microtubule associated protein (MAP2)	Polyclonal rabbit	Millipore, Billerica, MA, USA	1:500
Phospho-S6 ribosomal protein (pS6)	Polyclonal rabbit (Ser235/236; pS6)	Cell Signaling Technology, Beverly, MA, USA;	1:50
CD68	Mouse clone PG-M1	DAKO, Glostrup, Denmark	1:200
CD31	Mouse clone JC70A	DAKO, Glostrup, Denmark	1:10
Albumin	Rabbit polyclonal	DAKO, Glostrup, Denmark	1:20,000
Granzyme B (GrB)	Mouse clone GrB-7	Monosan, Uden, The Netherlands	1:100

For double-labeling of MHC-I with GFAP, MAP-2 or pS6 (as well as of albumin with CD31 or GFAP and GrB with GFAP) sections were, after incubation with the primary antibodies overnight at 4°C, incubated for 2 h at room temperature with Alexa Fluor® 568-conjugated anti-rabbit and Alexa Fluor® 488 anti-mouse immunoglobulin G (IgG) or anti-goat IgG (Molecular Probes, The Netherlands; 1:100). Sections were then analyzed using a laser scanning confocal microscope (Leica TCS Sp2, Wetzlar, Germany).

For double-labeling of MHC-I with NeuN, sections were incubated with the first primary antibody. Anti-MHC-I was visualized with a polymer-alkaline phosphatase (AP)-labeled anti-rabbit antibody (BrightVision #DPVM55AP, Immunologic, Duiven, The Netherlands) and Vector Red (AP substrate kit III, #SK-5100, Vector labs, Burlingame, CA, USA) as the chromogen. To remove the first primary antibody (MHC-I), the sections were incubated at 121°C in a citrate buffer (0.01 M, pH 6.0) for 10 min. Sections were then incubated for 1 h at room temperature with the second primary antibody (NeuN). The second primary antibodies were visualized with poly-AP anti-rabbit antibody (BrightVision #DPVM55AP, Immunologic, Duiven, The Netherlands) and Vector Blue (AP substrate kit III, #SK-5300, Vector labs, Burlingame, CA, USA) as the chromogen.

### Evaluation of immunostaining

**Figure 1:**
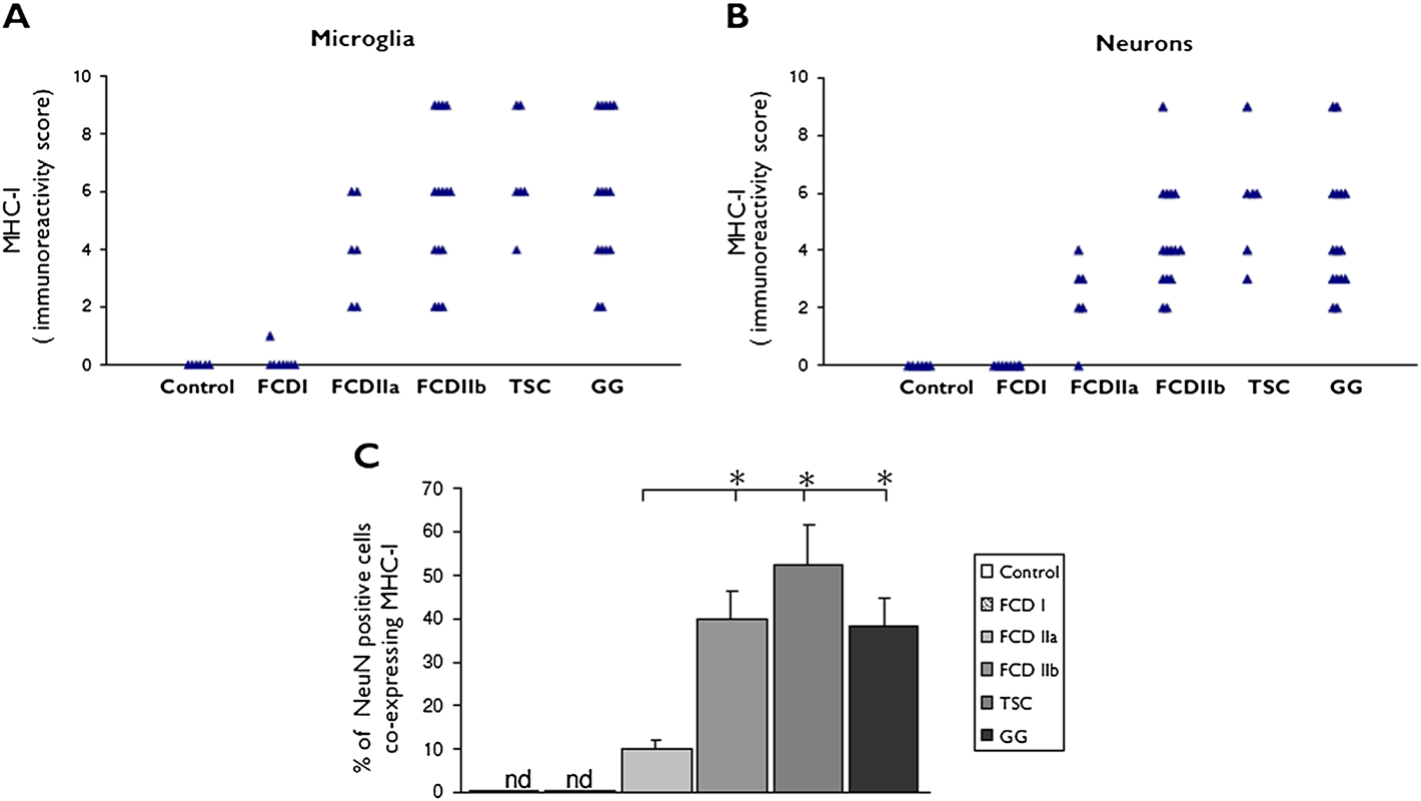
**Evaluation of MHC-I expression in control and FCD I, FCD IIa, FCD IIb, TSC and GG. (A)** and **(B)**: MHC-I immunoreactivity scores (total score; see material and methods for details) in microglial cells and neurons. **(C)**: Graph showing percentages of neuronal cells immunoreactive for NeuN and co-expressing MHC-I. Data are expressed as mean ± standard error of mean (SEM). *P* < 0.05 compared to FCD I and controls. FCD, focal cortical dysplasia; TSC, tuberous sclerosis complex; GG, ganglioglioma; MHC-I, major histocompatibility complex class I; ND, not determined; nd, not detectable.

**Figure 2:**
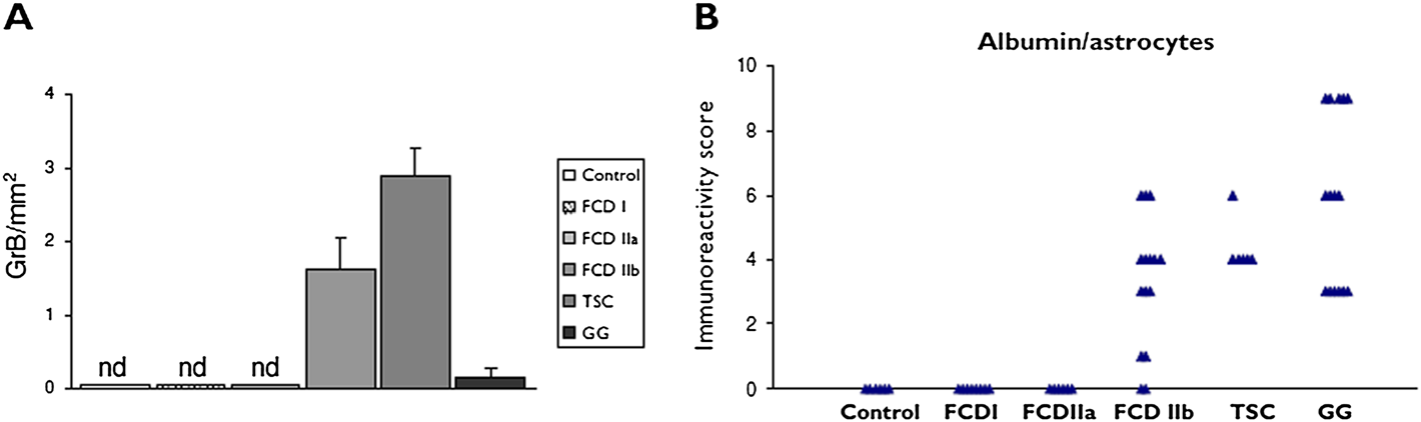
**Evaluation of GrB positive cells and albumin immunoreactivity (IR) in astrocytes. (A)**: Graph showing GrB cell counting in control cortex, FCD I, FCD IIa, FCD IIb and GG. * *P* < 0.05, compared to GG, FCD I, FCD IIa and controls. **(B)**: Albumin immunoreactivity scores (total score; see material and methods for details) in astrocytes.

All labeled tissue sections were evaluated by two independent observers blind to clinical data, for the presence or absence of various histopathological parameters and specific immunoreactivity for the different markers. Two representative sections per case were stained and assessed for the MHC-I and albumin. The intensity of the staining was evaluated as previously described [6,14], using a semi-quantitative scale ranging from 0 to 3 (0: negative; 1: weak; 2: moderate; 3: strong immunoreactivity). All areas of the specimen were examined and the score represents the predominant cell staining intensity found in each case. The approximate proportion of cells (microglia, neurons and endothelial cells/blood vessels) showing MHC-I immunoreactivity (1: single to 10%; 2: 11% to 50%; 3: >50%) was also scored to give information about the relative number (‘frequency’ score) of positive cells within the specimens with a malformation of cortical development. In the case of a disagreement, independent reevaluation was performed by both observers to define the final score. As proposed before [6,15], the product of these two values (intensity and frequency scores) was taken to give the overall score (immunoreactivity total score), which is shown in Figure 1 (MHC-I) and Figure 2 (albumin). Neuronal cell bodies were differentiated from glia and glia-neuronal balloon cells or giant cells based on morphology.

To analyze the percentage of double-labeled cells positive for MHC-I and NeuN, digital images of eight representative fields per section (magnification 20 times) were collected (Leica DM5000B). Images were analyzed with a Nuance VIS-FL Multi-spectral Imaging System (Cambridge Research Instrumentation; Woburn, MA) as previously described [16,17]. The total number of cells stained with MHC-I or NeuN, as well as the number of cells double labeled with both were counted visually and percentages were calculated (expressed as mean ± SEM) of cells co-expressing NeuN and MHC-I.

Quantitative analysis was performed for GrB and the numbers of positive cells were quantified as previously described [15,18]. Quantitative analysis of the staining intensity in endothelial cells was also performed. The relative optical density ratio (ODR) of endothelial cells immunolabeled with MHC-I was calculated as previously described [19]. The degree of MHC-I expression in microvessels was evaluated by counting the numbers of vessels expressing the protein in two non-overlapping microscopic fields (field size 1 mm^2^) of control and FCD IIb specimens (*n* = 6 in each group). Results were expressed as a normalized mean ± SEM of MHC-I positive vessels per microscopic field, taking into account the total number of microvessels in control and FCD specimens, assessed by counting the number of CD31-positive vessels in adjacent serial sections, as previously described [20].

### Statistical analysis

Statistical analyses were performed with SPSS for Windows (SPSS 11.5, SPSS Inc., Chicago, IL, USA). The two-tailed Student’s t-test was used to assess differences between groups. To assess differences between more than two groups a non-parametric Kruskal–Wallis test followed by a Mann–Whitney U test were used. Correlation between immunostaining (number of positive cells) and different clinical variables (duration of epilepsy, seizure frequency, age at surgery, age at seizure onset and epilepsy outcome) were assessed using the Spearman’s rank correlation test. A value of *P* < 0.05 was defined as being statistically significant.

## Results

### Case material and histological features

The clinical features of the cases included in this study are summarized in Table 1. All patients had a history of chronic pharmaco-resistant epilepsy. Age at surgery, seizure duration and seizure frequency were not statistically different between patients with FCD I, FCD II and GG in this cohort. Postoperatively, 67% of patients in this cohort were completely seizure free. In this study, we excluded patients with a mild degree of cortical dysplasia (mild malformation of cortical development). The FCD cases included displayed the histopathological features of FCD Ib or FCD IIa and IIb, according to the international consensus classification [5].

The histopathological features of the cortical tuber samples included cortical dyslamination, giant cells, dysplastic neurons and astrogliosis [21,22]. TSC1 mutations were detected in one patient and TSC2 mutations in five patients. In agreement with previous studies [6,23-25], the expression of pS6 (indicating the activation of the mTOR signal transduction pathway) was observed within all tubers, and FCD II and GG samples in our cohort. All of the 15 FCD IIb samples had a neuronal labeling index (frequency score) greater than 50%. Of the 6 FCD IIa samples with pS6-positive neuronal cells, 3 (50%) had a labeling index between 1% and 10%, and the other 3 (50%) had a labeling index between 11% and 50%. In contrast, pS6 protein expression was not detected in FCD I cases, as in the normal control specimens. The number of HLA-DR (MHC-II) immunoreactive cells was not significantly different between FCD IIa and IIb in our cohort, but was higher compared to FCD I, as previously reported [6].

### MHC-I expression in FCD I and II

**Figure 3:**
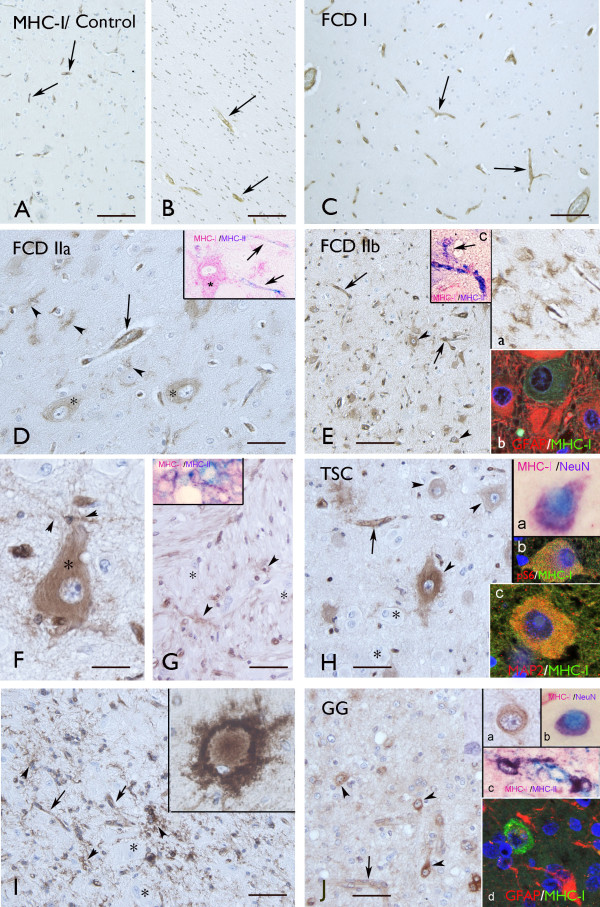
**Distribution of MHC-I immunoreactivity (IR) in FCD, TSC and GG.** (**A**)-(**B**) Control cortex (**A**) and white matter (wm; **B**): MHC-I IR in blood vessels (arrows). (**C**) FCD I: MHC-I IR in blood vessels (arrows). (**D**) FCD IIa: MHC-I IR in blood vessels (arrows), dysmorphic neurons (DN; asterisks) and microglial cells (arrowheads). Insert in (**D**) shows a neuron (asterisk) positive for MHC-I and both MHC-I and MHC-II IR in blood vessels (arrows). (**E**)–(**G**) FCD IIb: strong MHC-I IR in cortex, (**E**) and (**F**) and wm, (**G**) with expression in DN (arrowheads in (**E**) and asterisk in (**F**)). MHC-I IR was also detected in blood vessels (arrows in (**E**)) and in microglial cells (insert (**a**) in (**E**), arrowheads in (**F**) and (**G**)). The balloon cells are negative (asterisks in (**G**)). Insert (**b**) in (**E**): absence of co-localization of MHC-I with GFAP in astrocytes. Insert (**c**) in (**E**) and the insert in (**G**): co-localization of MHC-I with MHC-II in microglial cells (the arrow in (**c**) indicates a microglial cell). **(H), (I)** TSC: strong MHC-I IR in both cortex (**H**) and wm (**I**) with expression in DN (arrowheads in (**H**)), blood vessels (arrows in (**H**) and (**I**)) and microglia (arrowhead in (**I**)). Giant cells are negative (asterisks). MHC-I IR is occasionally detected in giant cells (insert in (**I**)). Insert (a) in (**H**): co-localization with NeuN. Insert (**b**) in (**H**): co-localization with pS6. Insert (**c**) in (**H**): co-localization with MAP-2. **(J)** GG: MHC-I IR in dysplastic neurons (arrowheads; high magnification in insert (**a**)) and blood vessels (arrow). Insert (**b**) in (**J**): co-localization with NeuN. Insert (**c**) in (J): co-localization with MHC-II in microglial cells. Insert (**d**) in (**J**): absence of co-localization with GFAP. Scale bars: (**A**)–(**C**) and (**E**): 150 μm; (**D**), (**G**)–(**I**): 40 μm; (**F**): 25 μm; (**J**): 80 μm.

In the cortical autopsy of the human control, as well as in the normal-appearing cortex adjacent to the lesions (not shown), MHC-I was confined to blood vessels, and was not detected either in neurons or in glial cells in both cortex and white matter throughout all cortical layers (Figure 3A,B).

In agreement with previous observations, in both viral [13] and Rasmussen’s encephalitis [8,9] immunoreactivity for MHC-I was also detected in lymphocytes, microglial cells, astrocytes and neurons (not shown).

In FCD I, MHC-I displayed an immunoreactivity pattern as in the controls, with expression in blood vessels, but no detectable immunoreactivity in glial or neuronal cells (Figure 3C and Figure 1A,B); in one case weak immunoreactivity was detected in a few microglial cells (Figure 1A).

In FCD IIa and IIb, consistent MHC-I expression was also observed in neurons and glial cells with the morphology of microglial cells (Figure 3D,E,F,G and Figure 1A,B). Double labeling confirmed MHC-I expression in cells of the microglial/macrophage lineage (HLA-DR positive cells; Figure 3 and inserts in 3E and 3G), in neurons (MAP-2 and NeuN positive cells; not shown), but not in astrocytes (Figure 3; insert (b) in 3E). The mean number of MHC-I positive neurons was found to be significantly higher in FCD IIb than in FCD IIa, whereas no significant differences were detected compared to TSC and GG specimens (Figure 1C). We did not detect MHC-I expression in balloon cells (Figure 3E,G). MHC-I immunoreactivity in microvessel endothelium was not significantly different compared to the control cortex (autopsy; Table 3, and/or the perilesional cortex, not shown). No significant correlation was found between the increased MHC-I staining in neurons in FCD II and the different clinical variables. However, a positive correlation was detected between the MHC-I staining in microglia and the duration of epilepsy (Spearman’s rank correlation coefficient for FCD II, *r* = 0.865 with *P* < 0.05).

**Table 3:** **MHC-I immunoreactivity in microvessel endothelium**^**a**^

	**Control (*n *= 6)**	**FCD IIb (*n *= 6)**
Intensity score	1.47 ± 0.06	1.52 ± 0.10
ODR	2.8 ± 0.3	3.1 ± 0.4

^a^Scoring of the histological specimens was performed as described in the Materials and methods section. Values represent the mean ± SEM of the number of samples indicated in parentheses. ODR: relative optical density ratio of CD31-immunolabeled endothelium.

### MHC-I expression in cortical tubers (TSC)

The expression pattern of MHC-I in cortical tubers of TSC patients resembled that observed in FCD II, with MHC-I positive blood vessels, neurons and microglial cells, but not astrocytes (Figure 3H,I and Figure 1A,B). The mean number of MHC-I positive neurons was found to be significantly higher in TSC than in FCD IIa, whereas no significant differences were detected compared to FCD IIb and GG specimens (Figure 1C). A large majority of giant cells were negative for MHC-I, only occasionally (in two cases) was MHC-I expression detected in sporadic giant cells within the white matter (Figure 3I). No significant correlation was found between the increased MHC-I staining in neurons in TSC and the different clinical variables. However, a positive correlation was detected between the MHC-I staining in microglia in TSC and the duration of epilepsy (Spearman’s rank correlation coefficient for TSC, *r* = 0.835 with *P* < 0.05).

### MHC-I expression in GG

In GG specimens, MHC-I immunoreactivity was detected in blood vessels, neurons and microglial cells, but not in tumor astrocytes (Figure 3J and Figure 1A,B). The mean number of MHC-I positive neurons was found to be significantly higher in GG than in FCD IIa, whereas no significant differences were detected compared to FCD IIb and TSC specimens (Figure 1C).

No significant correlation was found between the increased MHC-I staining in neurons in GG and the different clinical variables. However, a positive correlation was detected between the MHC-I staining in microglia and the duration of epilepsy (Spearman’s rank correlation coefficient for GG, *r* = 0.847 with *P* < 0.05).

### T lymphocytes with granzyme B^+^ granules

**Figure 4:**
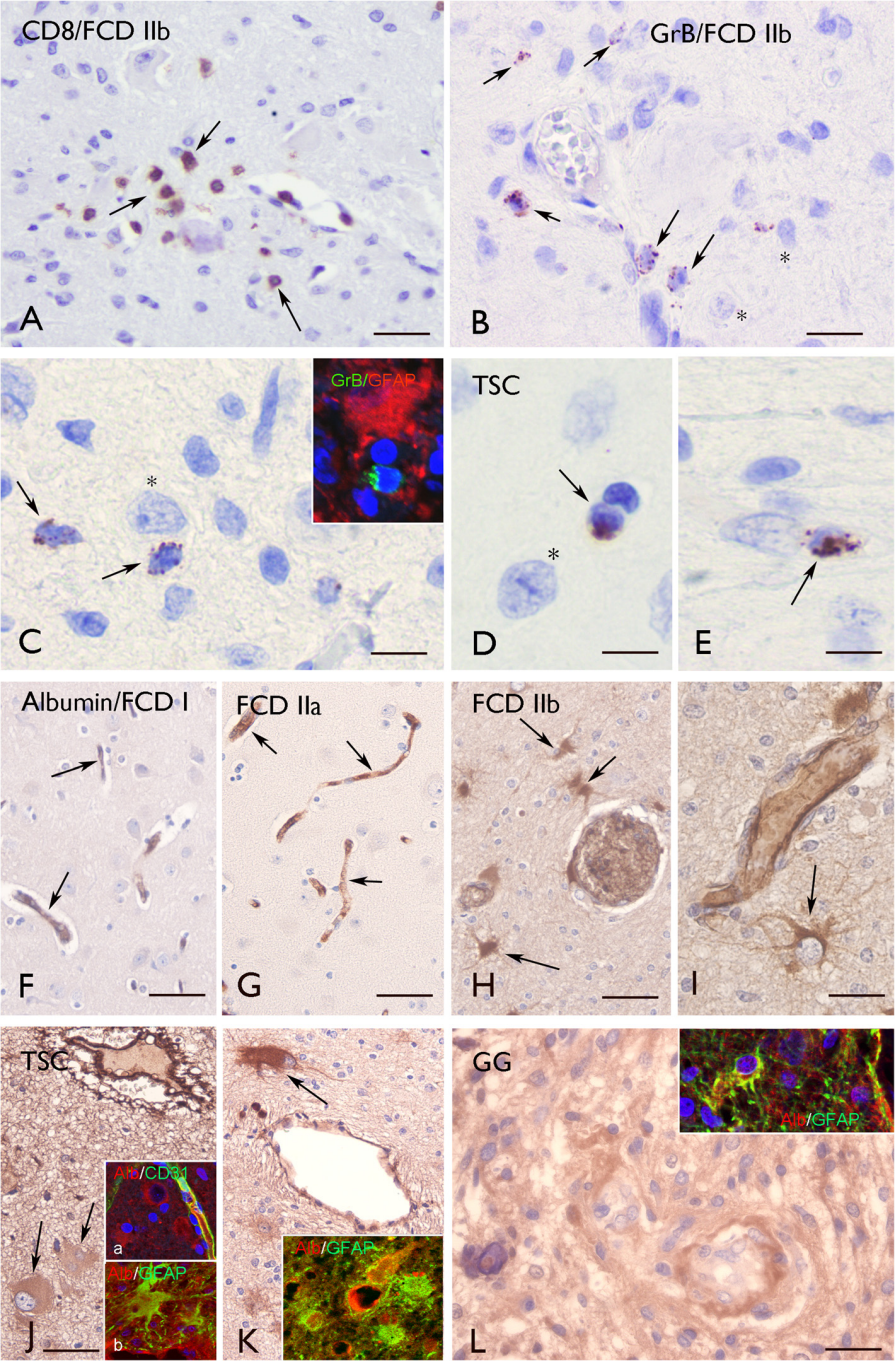
**Granzyme B (GrB) and albumin immunoreactivity (IR).** (**A**)–(**E**) Granzyme B (GrB) IR. (**A**) Perivascular CD8 positive cells (arrows) for FCD IIb. (**B**) and (**C**) GrB positive cells (arrows) for FCD IIb around blood vessels and in the vicinity of dysmorphic neurons (asterisks). Insert in (**C**) shows a GrB positive cell near an astrocyte (GFAP positive). (**D**) and **(E**) GrB positive cells in cortical tubers (arrows) for TSC, also the vicinity of a dysmorphic neuron (asterisk in (**D**)). (**F**)–(**L**) Albumin IR. For FCD I (**F**) and FCD IIa (**G**) there is an absence of glial IR. Albumin IR was detected in blood vessels (arrows). (**H**) and (**I**) FCD IIb: Albumin IR was detected in perivascular astrocytes (arrows). (**J**) and (**K**) TSC: Strong albumin IR was detected within the tuber in the perivascular region with expression in giant cells (arrows). Insert (**a**) in (**J**) shows albumin in cells surrounding a blood vessel (stained with CD31). Insert (**b**) in (**J**) and the insert in (**K**) show co-localization of albumin with GFAP within tubers. (**L**) For GG, there is strong albumin IR within the tumor. The insert shows co-localization of albumin with GFAP in tumor astrocytes. Scale bars (**A**): 40 μm; (**B**): 25 μm; (**C**)–(**E**): 20 μm; (**F**)–(**H**), (**J**) and (**K**) (bar in (**J**)): 80 μm; (**I**), (**L**): 40 μm.

In agreement with previous observations [6,26,27], inflammatory infiltrates in glioneuronal lesions, such as FCD IIb, contain T lymphocytes (CD8^+^, T-cytotoxic immunophenotype) (Figure 4A).

In FCD IIb and TSC, we detected CD8 positive cells with granzyme-B^+^ granules. These cells were often perivascularly located, but occasionally in the vicinity (or in close opposition) to dysmorphic neurons and astrocytes (Figure 4B,C,D,E and Figure 2A). Granzyme B immunoreactivity was not detected in FCD I or IIa, and only occasionally (one case) in GG specimens (Figure 2A).

### Albumin extravasation and immunoreactivity in astrocytes

Alterations in blood–brain barrier permeability were detected using albumin immunocytochemistry in FCD IIb, TSC and GG specimens (Figure 4H,I,J,K,L and Figure 2B). In FCD IIb and TSC specimens, albumin immunoreactivity, with diffuse cytoplasmatic staining, was detected in perilesional astrocytes and in perivascularly located balloon/giant cells in FCD IIb and TSC specimens (Figure 4H,I,J,K); double labeling confirmed co-localization in GFAP positive cells (Figure 4J,K). Strong albumin immunoreactivity was observed within GG specimens in tumor astrocytes (Figure 4L and Figure 2B).

## Discussion

In the present study we provide evidence of neuronal and microglial MHC-I expression in epileptogenic glioneuronal lesions. The neuronal expression of MHC-I was, however, only detected in FCD II, but not in FCD I specimens or in the perilesional region (despite the absence of significant differences in seizure frequency and duration). Interestingly, MHC-I expression in neurons was significantly higher in FCD IIb compared to FCD IIa and the large majority of balloon/giant cells did not express detectable levels of MHC-I. These observations confirm the difference between FCD I and II [6], suggest some differences between IIa and IIb and indicate that induction of MHC-I is not simply an effect of seizure activity.

Several studies demonstrate that expression of MHC-I can be upregulated in glia and neurons in response to different types of challenges, including injury, infections (chronic and acute), central administration of endotoxins and exposure to different cytokines ( [28-33] reviewed in [7]). Cytokines have been show to differentially regulate MHC-I induction in neurons [31,34,35]. Previous studies have demonstrated prominent expression of components of the IL-1R/TLR signaling pathways in neuronal cells in epileptogenic glioneuronal lesions [6,26,27,36]. Signaling through these pathways leads to activation of the transcription factor, nuclear factor-kappa B (NF-κB) [37]. Interestingly, it has been suggested that activation of NF-κB plays a role in induction of MHC-I [38,39]. Thus, NF-κB-dependent mechanisms of regulation may contribute to the more prominent MHC-I expression detected in FCD II (associated with consistent activation of IL-1R/TLR signaling pathways [6]) compared to FCD I.

Endogenous peptides presented by MHC-I molecules (called MHC-I peptides (MIPs) or the immunopeptidome) represent the key to self/non-self-discrimination by cells of the immune system. In this respect, a recent study confirmed previous observations indicating that the immune system is tolerant to MIPs expressed at physiological levels but may promote immune responses towards self MIPs present in excessive amounts [40,41]. This may be important in the context, of the suggestion that neuronal MHC-I expression mediates removal of dysfunctional neurons [30]. The study by Caron *et al*. also suggests that changes in mTOR signaling can affect the expression of MHC-I and the repertoire of MIPs presented by MHC-I [41]. These observations highlight the complexity of MHC-I regulation and indicate the need for further analysis of the effect of mTOR modulation in lesions (such as FCD II, TSC and GG) in which this pathway is involved. Interestingly, a recent study suggests a novel viral etiology for FCD IIB [42], which could explain the constitutive activation of mTOR, as well as the induction of MHC-I, in this focal malformation of cortical development.

Interestingly, neuronal MHC-I expression has been reported in Rasmussen’s encephalitis and it has been suggested that it plays a critical role in antigen-specific cytotoxicity [8]. MHC-I expression is necessary for antigen-specific cytotoxicity mediated by CD8^+^ lymphocytes [43]. In this context, we also detected CD8^+^ T lymphocytes with GrB in FCD IIB and TSC specimens in the vicinity of neurons. The possible contribution of a MHC-I restricted immune response to neuronal injury, occurring in patients with developmental pathologies and intractable epilepsy [18,44], requires further investigation.

MHC-I was also was found to be expressed in reactive microglial cells. Upregulation in microglial cells has been shown in both multiple sclerosis (MS) [45] and Rasmussen’s encephalitis [8,9]. Interestingly, the microglial expression of MHC-I was significantly higher in FCD II compared to FCD I, reflecting the more prominent activation of microglial cells observed in FCD II specimens [6]. Moreover, MHC-I expression in microglia correlated positively with the duration of epilepsy, suggesting that the upregulation of MHC-I in these cells may also occur later in epileptogenesis.

Astrocytes did not display MHC-I immunoreactivity. Expression of MHC-I in astrocytes has also not been observed in MS lesions [45]. Thus upregulation of MHC-I in astrocytes appears to represent a specific feature of Rasmussen’s encephalitis and an MHC-I restricted T-cell response has been suggested as critically contributing to the occurrence of the astrocytic degeneration observed in this pathology [9].

MHC-I expression was not detected in balloon cells in FCD IIb and in the large majority of giant cells in TSC. Interestingly, expression of MHC-I has been detected in the giant cells from different types of TSC-associated brain lesions in fetal cases ranging from 23 to 34 gestational weeks (GW) [46], indicating developmental changes in the phenotype of giant cells. MHC-I expression in these cells early in development may reflect their role as antigen-presenting cells and may account for the dynamic changes occurring early in development in TSC lesions.

We did not detect changes in the expression levels of MHC-I in endothelial cells within the different lesions examined. However, lesions (FCD IIB, TSC and GG) with prominent inflammatory changes and MHC-I upregulation in neurons and microglia displayed evidence of alterations in blood–brain barrier permeability, with albumin extravasation and uptake in astrocytes. These observations confirm previous findings for TSC [26], highlighting its similarity to FCD IIB and the differences with FCD I.

Our findings distinguish type I from type II FCD and indicate a prominent upregulation of MHC-I in neurons and microglial cells as part of the immune response occurring in epileptogenic glioneuronal lesions, such as FCD II, TSC and GG.

## Abbreviations

AP: alkaline phosphatase; FCD: focal cortical dysplasia; GFAP: glial fibrillary acidic protein; GG: ganglioglioma; GrB: granzyme B; HLA: human leukocyte antigen; IgG: immunoglobulin G; IR: immunoreactivity; MHC: major histocompatibility complex; MIP: MHC-I peptide; MS: multiple sclerosis; NF-κB: nuclear factor-kappa B; NeuN: neuronal nuclear protein; ODR: optical density ratio; SEM: standard error of mean; TSC: tuberous sclerosis complex.

## Competing interest

None of the authors has any conflict of interest to disclose.

## Authors’ contributions

Immunohistochemistry and analysis of the data were performed by AP, AI and JA. EA helped with the analysis of the data. AI and AP helped EA in drafting and preparing the manuscript for submission. The overall experimental design was conceived and supervised by EA. WS and PR helped in the selection and collection of brain tissues. All authors read and approved the final manuscript.

## References

[B1] Aronica E Crino PB Inflammation in epilepsy: clinical observations Epilepsia 2011 52 Suppl 3 26 32 21542843 10.1111/j.1528-1167.2011.03033.x

[B2] Vezzani A French J Bartfai T Baram TZ The role of inflammation in epilepsy Nature Reviews Neurology 2011 7 31 40 10.1038/nrneurol.2010.178 PMC3378051 21135885

[B3] Vezzani A Auvin S Ravizza T Aronica E Noebels JL, Avoli M, Rogawski MA, Olsen RW, Delgado-Escueta AV Glia-neuronal interactions in ictogenesis and epileptogenesis: role of inflammatory mediators Jasper’s Basic Mechanisms of the Epilepsies 2012 4 Bethesda, MD 22787662

[B4] Aronica E Ravizza T Zurolo E Vezzani A Astrocyte immune responses in epilepsy Glia 2012 60 1258 1268 10.1002/glia.22312 22331574

[B5] Blumcke I Thom M Aronica E Armstrong DD Vinters HV Palmini A Jacques TS Avanzini G Barkovich AJ Battaglia G The clinicopathologic spectrum of focal cortical dysplasias: a consensus classification proposed by an ad hoc Task Force of the ILAE Diagnostic Methods Commission Epilepsia 2011 52 158 174 10.1111/j.1528-1167.2010.02777.x 21219302 PMC3058866

[B6] Iyer A Zurolo E Spliet WGM van Rijen PC Baayen JC Gorter JA Aronica E Evaluation of the innate and adaptive immunity in type I and type II focal cortical dysplasias Epilepsia 2010 51 9 1763 1773 10.1111/j.1528-1167.2010.02547.x 20345941

[B7] Boulanger LM MHC class I in activity-dependent structural and functional plasticity Neuron Glia biology 2004 1 283 289 18185853 10.1017/S1740925X05000128 PMC2184613

[B8] Bien CG Bauer J Deckwerth TL Wiendl H Deckert M Wiestler OD Schramm J Elger CE Lassmann H Destruction of neurons by cytotoxic T cells: a new pathogenic mechanism in Rasmussen’s encephalitis Ann Neurol 2002 51 311 318 10.1002/ana.10100 11891826

[B9] Bauer J Elger CE Hans VH Schramm J Urbach H Lassmann H Bien CG Astrocytes are a specific immunological target in Rasmussen’s encephalitis Ann Neurol 2007 62 67 80 10.1002/ana.21148 17503512

[B10] Engel JJ Engel JJ Outcome with respect to epileptic seizures Surgical Treatment of the Epilepsies 1993 New York: Raven Press 609 621

[B11] Gomez M Sampson J Whittemore V The Tuberous Sclerosis Complex 1999 Oxford: Oxford University Press

[B12] Louis DN Ohgaki H Wiestler OD Cavanee WK WHO Classification of Tumours of the Central Nervous System 2007 Lyon: IARC 10.1007/s00401-007-0243-4 PMC1929165 17618441

[B13] van Thiel PP de Bie RM Eftimov F Tepaske R Zaaijer HL van Doornum GJ Schutten M Osterhaus AD Majoie CB Aronica E Fatal human rabies due to Duvenhage virus from a bat in Kenya: failure of treatment with coma-induction, ketamine, and antiviral drugs PLoS Neglected Tropical Diseases [electronic resource] 2009 3 428 10.1371/journal.pntd.0000428 PMC2710506 19636367

[B14] Ravizza T Boer K Redeker S Spliet WG van Rijen PC Troost D Vezzani A Aronica E The IL-1beta system in epilepsy-associated malformations of cortical development Neurobiol Dis 2006 24 128 143 10.1016/j.nbd.2006.06.003 16860990

[B15] Aronica E Gorter JA Redeker S Ramkema M Spliet WG van Rijen PC Leenstra S Troost D Distribution, characterization and clinical significance of microglia in glioneuronal tumours from patients with chronic intractable epilepsy Neuropathol Appl Neurobiol 2005 31 280 291 10.1111/j.1365-2990.2004.00636.x 15885065

[B16] van der Loos CM Multiple immunoenzyme staining: methods and visualizations for the observation with spectral imaging J Histochem Cytochem 2008 56 313 328 18158282 10.1369/jhc.2007.950170 PMC2326109

[B17] Boer K Troost D Timmermans W Gorter JA Spliet WG Nellist M Jansen F Aronica E Cellular localization of metabotropic glutamate receptors in cortical tubers and subependymal giant cell tumors of tuberous sclerosis complex Neuroscience 2008 156 203 215 10.1016/j.neuroscience.2008.06.073 18706978

[B18] Maldonado M Baybis M Newman D Kolson DL Chen W McKhann G 2nd Gutmann DH Crino PB Expression of ICAM-1, TNF-alpha, NF kappa B, and MAP kinase in tubers of the tuberous sclerosis complex Neurobiol Dis 2003 14 279 290 10.1016/S0969-9961(03)00127-X 14572449

[B19] Aronica E Gorter JA Redeker S van Vliet EA Ramkema M Scheffer GL Scheper RJ van der Valk P Leenstra S Baayen JC Localization of breast cancer resistance protein (BCRP) in microvessel endothelium of human control and epileptic brain Epilepsia 2005 46 849 857 10.1111/j.1528-1167.2005.66604.x 15946326

[B20] Airas L Lindsberg PJ Karjalainen-Lindsberg ML Mononen I Kotisaari K Smith DJ Jalkanen S Vascular adhesion protein-1 in human ischaemic stroke Neuropathol Appl Neurobiol 2008 34 394 402 10.1111/j.1365-2990.2007.00911.x 18005095

[B21] Mizuguchi M Takashima S Neuropathology of tuberous sclerosis Brain Dev 2001 23 508 515 10.1016/S0387-7604(01)00304-7 11701246

[B22] DiMario FJ Jr Brain abnormalities in tuberous sclerosis complex J Child Neurol 2004 19 650 657 15563010 10.1177/08830738040190090401

[B23] Baybis M Yu J Lee A Golden JA Weiner H McKhann G 2nd Aronica E Crino PB mTOR cascade activation distinguishes tubers from focal cortical dysplasia Ann Neurol 2004 56 478 487 10.1002/ana.20211 15455405

[B24] Schick V Majores M Engels G Hartmann W Elger CE Schramm J Schoch S Becker AJ Differential Pi3K-pathway activation in cortical tubers and focal cortical dysplasias with balloon cells Brain Pathology 2007 17 165 173 10.1111/j.1750-3639.2007.00059.x 17388947 PMC8095540

[B25] Boer K Troost D Timmerman W Spliet WGM van Rijen PC Aronica E Pi3K-mTOR signaling and AMOG expression in epilepsy-associated glioneuronal tumors Brain Pathology 2010 20 234 244 10.1111/j.1750-3639.2009.00268.x 19371356 PMC8094642

[B26] Boer K Jansen F Nellist M Redeker S van den Ouweland AM Spliet WG van Nieuwenhuizen O Troost D Crino PB Aronica E Inflammatory processes in cortical tubers and subependymal giant cell tumors of tuberous sclerosis complex Epilepsy Res 2008 78 7 21 10.1016/j.eplepsyres.2007.10.002 18023148

[B27] Aronica E Boer K Becker A Redeker S Spliet WG van Rijen PC Wittink F Breit T Wadman WJ Lopes da Silva FH Gene expression profile analysis of epilepsy-associated gangliogliomas Neuroscience 2008 151 272 292 10.1016/j.neuroscience.2007.10.036 18093740

[B28] Maehlen J Schroder HD Klareskog L Olsson T Kristensson K Axotomy induces MHC class I antigen expression on rat nerve cells Neurosci Lett 1988 92 8 13 10.1016/0304-3940(88)90733-1 3185980

[B29] Pereira RA Tscharke DC Simmons A Upregulation of class I major histocompatibility complex gene expression in primary sensory neurons, satellite cells, and Schwann cells of mice in response to acute but not latent herpes simplex virus infection in vivo J Exp Med 1994 180 841 850 10.1084/jem.180.3.841 8064236 PMC2191654

[B30] Neumann H Cavalie A Jenne DE Wekerle H Induction of MHC class I genes in neurons Science 1995 269 549 552 10.1126/science.7624779 7624779

[B31] Neumann H Schmidt H Cavalie A Jenne D Wekerle H Major histocompatibility complex (MHC) class I gene expression in single neurons of the central nervous system: differential regulation by interferon (IFN)-gamma and tumor necrosis factor (TNF)-alpha J Exp Med 1997 185 305 316 10.1084/jem.185.2.305 9016879 PMC2196130

[B32] Kimura T Griffin DE The role of CD8(+) T cells and major histocompatibility complex class I expression in the central nervous system of mice infected with neurovirulent Sindbis virus J Virol 2000 74 6117 6125 10.1128/JVI.74.13.6117-6125.2000 10846095 PMC112110

[B33] Foster JA Quan N Stern EL Kristensson K Herkenham M Induced neuronal expression of class I major histocompatibility complex mRNA in acute and chronic inflammation models J Neuroimmunol 2002 131 83 91 10.1016/S0165-5728(02)00258-8 12458039

[B34] Neumann H Control of glial immune function by neurons Glia 2001 36 191 199 10.1002/glia.1108 11596127

[B35] Neumann H The immunological microenvironment in the CNS: implications on neuronal cell death and survival J Neural Transm Suppl 2000 59 59 68 10961419 10.1007/978-3-7091-6781-6_9

[B36] Boer K Crino PB Gorter JA Nellist M Jansen FE Spliet WG van Rijen PC Wittink F Breit T Troost D Gene expression analysis of tuberous sclerosis complex cortical tubers reveals increased expression of adhesion and inflammatory factors Brain Pathol 2010 20 704 719 19912235 10.1111/j.1750-3639.2009.00341.x PMC2888867

[B37] Oeckinghaus A Hayden MS Ghosh S Crosstalk in NF-kappaB signaling pathways Nat Immunol 2011 12 695 708 21772278 10.1038/ni.2065

[B38] Kesson AM Cheng Y King NJ Regulation of immune recognition molecules by flavivirus, West Nile Viral immunology 2002 15 273 283 10.1089/08828240260066224 12081012

[B39] Forloni M Albini S Limongi MZ Cifaldi L Boldrini R Nicotra MR Giannini G Natali PG Giacomini P Fruci D NF-kappaB, and not MYCN, regulates MHC class I and endoplasmic reticulum aminopeptidases in human neuroblastoma cells Cancer Res 2010 70 916 924 10.1158/0008-5472.CAN-09-2582 20103633

[B40] Schild H Rotzschke O Kalbacher H Rammensee HG Limit of T cell tolerance to self proteins by peptide presentation Science 1990 247 1587 1589 10.1126/science.2321019 2321019

[B41] Caron E Vincent K Fortier MH Laverdure JP Bramoulle A Hardy MP Voisin G Roux PP Lemieux S Thibault P Perreault C The MHC I immunopeptidome conveys to the cell surface an integrative view of cellular regulation Mol Syst Biol 2011 7 533 21952136 10.1038/msb.2011.68 PMC3202804

[B42] Chen J Tsai V Parker WE Aronica E Baybis M Crino PB Detection of human papillomavirus in human focal cortical dysplasia type IIB Ann Neurology 2012 72 881 892 10.1002/ana.23795 23280839

[B43] Christinck ER Luscher MA Barber BH Williams DB Peptide binding to class I MHC on living cells and quantitation of complexes required for CTL lysis Nature 1991 352 67 70 10.1038/352067a0 2062379

[B44] Choi J Nordli DR Jr Alden TD DiPatri A Jr Laux L Kelley K Rosenow J Schuele SU Rajaram V Koh S Cellular injury and neuroinflammation in children with chronic intractable epilepsy J Neuroinflammation 2009 6 38 10.1186/1742-2094-6-38 20021679 PMC2811703

[B45] Gobin SJ Montagne L Van Zutphen M Van Der Valk P Van Den Elsen PJ De Groot CJ Upregulation of transcription factors controlling MHC expression in multiple sclerosis lesions Glia 2001 36 68 77 10.1002/glia.1096 11571785

[B46] Prabowo AS Anink JJ Lammens M Nellist M van den Ouweland AM Adle-Biassette H Sarnat HB Flores-Sarnat L Crino PB Aronica E Fetal brain lesions in tuberous sclerosis complex: TORC1 activation and inflammation Brain Pathology 2012 23 45 59 22805177 10.1111/j.1750-3639.2012.00616.x PMC3518755

